# Development of a novel drug for uncomplicated malaria targeting the mitochondrial NADH:quinone oxidoreductase

**DOI:** 10.1186/1475-2875-9-S2-O4

**Published:** 2010-10-20

**Authors:** Giancarlo A Biagini, Alasdair Hill, Alison Mbekeani, Alison Shone, Gemma Nixon, Paul Stocks, Peter Gibbons, Richard Amewu, David W Hong, Victoria Barton, Chandra Pidathala, James Chadwick, Louise Le Pensee, Ashley Warman, Raman Sharma, Nick Fisher, Neil G Berry, Paul M O'Neill, Steve A Ward

**Affiliations:** 1 Liverpool School of Tropical Medicine, Pembroke Place, Liverpool, L3 5QA, UK; 2 Department of Chemistry, Liverpool University, Pembroke Place, Liverpool, L3 5QA, UK

## 

NADH:quinone oxidoreductase (PfNDH2) represents a metabolic choke point in the respiratory chain of *Plasmodium falciparum* mitochondria and is the focus of a drug discovery programme. A miniaturised assay for recombinant PfNDH2 with robust assay performance measures was generated for the high throughput screening (HTS) of a focused library of 17,000 drug-like compounds. A quantitative structure-activity relationship has been developed around one of the chemical templates derived from the HTS hits. Lead molecules developed to date show selective inhibitory activity against PfNDH2 versus *P. falciparum* bc_1_ or dihydroorotate dehydrogenase (DHODH). Potent enzyme inhibition is accompanied by *in vitro* parasite kill of multidrug-resistant strains in the low nM range and clearance of parasites from *in vivo P. berghei* models. Lead molecules also display excellent *in vitro* therapeutic indices against human cell lines and bovine *bc1.* Initial metabolic studies in human liver microsomes and hepatocytes indicate favourable pharmacology. These data support the further development of this new candidate drug targeting a novel parasite component.

